# International Conference on Emerging Infectious Diseases

**DOI:** 10.3201/eid1011.040857

**Published:** 2004-11

**Authors:** Robert V. Tauxe, Rima F. Khabbaz, Daniel N. Cameron, Lori Feinman

**Affiliations:** * Centers for Disease Control and Prevention, Atlanta, Georgia, USA;; † American Society for Microbiology, Washington, D.C., USA

**Keywords:** ICEID Conference 2004, SARS

Approximately 2,100 scientists and public health officials from the United States and 64 other countries gathered in Atlanta, Georgia, February 29 – March 3, 2004, for the fourth biennial International Conference on Emerging Infectious Diseases. The aims of the conference were to present the latest scientific information from a variety of disciplines on the new and emerging microbial threats to public health around the world and to encourage and enhance the partnerships that are critical to addressing them. The conference was sponsored by the Centers for Disease Control and Prevention, the American Society for Microbiology, the Association of Public Health Laboratories, the Council of State and Territorial Epidemiologists, and the World Health Organization, as well as 38 partner organizations. The scientific program committee had representatives from 23 agencies and organizations.

The field of infectious diseases is fast-moving, as new challenges around the world engage the efforts of physicians, epidemiologists, microbiologists, veterinarians, and social scientists to understand them well enough to control and prevent them. Meeting these new challenges, with the best science and with the most effective policies, was the continuing theme of this multidisciplinary conference. The conference schedule was built around 12 plenary speakers and 16 panels of invited talks, along with 115 scientific oral presentations and 345 posters, chosen from 711 submitted abstracts. Four lunchtime sessions were devoted to discussing practical aspects of emergency response, such as the extensive experience with quarantine in the severe acute respiratory syndrome (SARS) epidemic. Six breakfast "meet-the-network" sessions introduced some of the current international surveillance and response networks. The International Conference on Women and Infectious Disease and other satellite meetings provided time for discussion and amplification of the interlocking issues and relationships.

The opening session outlined the central themes of the conference, with addresses by Julie Gerberding, director of the Centers for Disease Control and Prevention (CDC) and administrator of the Agency for Toxic Substances and Disease Registry; David Heymann, representing the director-general of the World Health Organization; William Sergeant, chairman of Rotary International's International PolioPlus Committee; and James Hughes, director of CDC's National Center for Infectious Diseases. The central themes they introduced recurred through many of the presentations. New pathogens, new antimicrobial resistance, and new routes of transmission continue to emerge as public health challenges. Many emerging pathogens are global, move swiftly from continent to continent, and cross readily from animal reservoirs to humans. Mounting an effective public health response depends on international collaboration across continents, cultures, and disciplines. Shaping effective mechanisms for control and prevention is the collective and exciting work of public health in the coming decade.

The conference also featured new information and major advances in understanding the latest global concerns, such as SARS, West Nile virus, avian influenza, and increasing antimicrobial resistance problems in many pathogens. The transformations of the developing world, with new patterns of consumption and leisure, are creating opportunities for the emergence of pathogens, at the same time as growing regional and international networks are evolving to better understand and monitor them. In many places, preparations to meet the threat of biocrime or bioterror are strengthening the capacity of public health systems to respond to future natural threats of pandemic and panzootic disease.

The conveners summarized presentations from each of the invited panels devoted to specific topics; some of these are published in this issue of Emerging Infectious Diseases. A tribute given at the conference to the late Robert Shope, a distinguished arbovirologist, has appeared in the journal (*1*). Planning has already begun on the fifth International Conference on Emerging Infectious Diseases, to be held in March 2006 in Atlanta.
